# Inflammation, Extracellular Matrix Remodeling, and Proteostasis in Tumor Microenvironment

**DOI:** 10.3390/ijms22158102

**Published:** 2021-07-28

**Authors:** Marina Marozzi, Arianna Parnigoni, Aide Negri, Manuela Viola, Davide Vigetti, Alberto Passi, Evgenia Karousou, Federica Rizzi

**Affiliations:** 1 Department of Medicine and Surgery, University of Parma, Via Gramsci 14, 43125 Parma, Italy; marina.marozzi@unipr.it (M.M.); aide.negri@unipr.it (A.N.); federicamariaangel.rizzi@unipr.it (F.R.); 2 Department of Medicine and Surgery, University of Insubria, Via J.H. Dunant 5, 21100 Varese, Italy; a.parnigoni@uninsubria.it (A.P.); manuela.viola@uninsubria.it (M.V.); davide.vigetti@uninsubria.it (D.V.); alberto.passi@uninsubria.it (A.P.)

**Keywords:** extracellular matrix remodeling, inflammation, tumor microenvironment, matrix metalloproteases, hyaluronan, extracellular chaperones, clusterin

## Abstract

Cancer is a multifaceted and complex pathology characterized by uncontrolled cell proliferation and decreased apoptosis. Most cancers are recognized by an inflammatory environment rich in a myriad of factors produced by immune infiltrate cells that induce host cells to differentiate and to produce a matrix that is more favorable to tumor cells’ survival and metastasis. As a result, the extracellular matrix (ECM) is changed in terms of macromolecules content, degrading enzymes, and proteins. Altered ECM components, derived from remodeling processes, interact with a variety of surface receptors triggering intracellular signaling that, in turn, cancer cells exploit to their own benefit. This review aims to present the role of different aspects of ECM components in the tumor microenvironment. Particularly, we highlight the effect of pro- and inflammatory factors on ECM degrading enzymes, such as metalloproteases, and in a more detailed manner on hyaluronan metabolism and the signaling pathways triggered by the binding of hyaluronan with its receptors. In addition, we sought to explore the role of extracellular chaperones, especially of clusterin which is one of the most prominent in the extracellular space, in proteostasis and signaling transduction in the tumor microenvironment. Although the described tumor microenvironment components have different biological roles, they may engage common signaling pathways that favor tumor growth and metastasis.

## 1. Introduction

Tumorigenesis is characterized by an uncontrolled proliferation of cells that have usually undergone genetic mutations due to hereditary, environmental, and lifestyle factors. Cancer progression, persistent inflammation of the surrounding tissue, and extracellular matrix (ECM) remodeling are three highly interconnected processes. Indeed, a multifaceted network of inflammatory signals produced by cancer cells and innate immune cells recruited in the tumor microenvironment (TME) induces changes in the surrounding stroma that, in turn, influence the homeostasis of ECM [1], creating a “cancerized” microenvironment that supports tumor growth and metastasis [2]. For instance, inflammatory cytokines and growth factors stimulate the trans-differentiation of stromal progenitors into cancer-associated fibroblasts (CAFs), a heterogeneous population of cells that are in dynamic evolution with the TME during cancer progression. CAFs, through autocrine and paracrine signaling, feed forward the inflammasome network that drives metastasis by enhancing angiogenesis, ECM remodeling, and epithelial-to-mesenchymal transition (EMT) [3].

The ECM represents a well-organized and heterogeneous network of macromolecules that provides a mechanical scaffold to the cells and mediates the diffusion of signaling molecules to sustain cell functions. Among the most important ECM macromolecules, hyaluronan (HA), which is a high molecular weight glycosaminoglycan (GAG), is produced by both stromal and tumor cells and has an active role in inflammatory cell functions in TME [4]. The structure of HA is simple and linear, it is neither sulfated nor linked to a protein core, and the only parameter that distinguishes the various roles of HA in physiological and pathological conditions is the molecular weight (see below). For instance, fragmentation of HA is involved in angiogenesis and inflammation, processes that characterize tumor progression. Various types of tumors, such as ovarian and breast, are characterized by an accumulation of HA that contributes to the invasion of cancer cells in both early and later stages of cancer, also predicting poor patient survival [5,6]. The altered cell functions in tumors are related to activated cell signaling pathways that are triggered by the binding of HA with cell surface receptors, such as CD44 and RHAMM. High levels of HA in cancer are usually associated with elevated levels of these receptors and, thus, many studies involve HA/receptor systems as a target in therapeutic strategies [7].

The invasion and metastasis of tumor cells are also supported by ECM remodeling provided by the degradation of the cells’ surrounding environment [2]. Enzymes that digest ECM proteins and GAGs have a double role in tumor progression. The first is to restructure the ECM architecture to one more susceptible to cell migration. The second is to generate fragments (matrikines) that bind to cell-surface receptors of the resident cells and trigger signals favoring the establishment of a cancer permissive TME. Studies in the literature, including clinical trials, are focused on the role of the high levels of matrix metalloproteases (MMPs), that degrade various types of proteins and collagens, and hyaluronidases, that degrade HA (see Section 3). Indeed, there is a dynamic correlation between MMPs levels and autocrine feedback that control their synthesis and activation [8].

Cancer cells are required to survive in an inflamed, acidified, hypoxic, and generally oxidized TME. Therefore, they are subjected to protein homeostasis impairment that may lead to proteotoxic stress. As an adaptive response to hostile environmental conditions, they upregulate the production of stress-activated proteins that reach the extracellular space by many different mechanisms. Once in the TME, these proteins act as extracellular chaperones to restore the protein homeostasis and take part in the tumor-stroma cross-talk by influencing critical aspects of cancer progression including ECM remodeling, surface receptors interaction, and signal transduction [9].

In this review, we aim to present the role of ECM in tumor progression, including the effect of inflammation-related secreted factors on the alterations of ECM composition. We describe the changes in ECM components and how these modified components, once linked to cell membrane receptors, may trigger signaling pathways that favor tumor cell survival. We intend to stress three main issues that coexist during cancer progression: (i) inflammation, (ii) ECM remodeling, with a special focus on HA metabolism, and (iii) the production of extracellular chaperones, as an adaptive response of cancer cells to survive in a hostile microenvironment. The whole picture that we present here aims to show how the three processes while remaining independent aspects of tumorigenesis, influence each other through complex signaling that takes place in the TME and pinpoints the active role played by ECM in tumor progression.

## 2. Inflammation and ECM Remodeling, Close Allies in Cancer Progression

In 1986, Dvorak defined cancers as “wounds that do not heal”. This definition comes from the observation that tumor growth leads to a continuous injury of the surrounding stromal tissue that triggers, in turn, a chronic response in the attempt to repair the lesion and restore tissue homeostasis [10]. This response is very complex and multifaceted and includes the concerted activity of inflammatory molecules, immune cells, and fibroblasts, which together lead to a state of persistent inflammation.

When a tumor lesion occurs, immune cells, as leukocytes, macrophages, and/or bone marrow-derived myeloid precursors, are recruited at the injured site. These tumor-infiltrating cells, along with cancer cells, release in the TME high levels of transforming growth factor β (TGFβ), a cytokine that exerts pleiotropic effects on both cancer and normal cells adjacent to rupture through autocrine and paracrine signaling mechanisms [11].

In normal and premalignant stages of cell transformation, TGFβ exerts tumor-inhibiting functions by suppressing cancer cell growth through anti-proliferative and pro-apoptotic signaling [12]. Moreover, TGFβ limits the proliferation and differentiation of cells of the innate and adaptive immune system, thus suppressing tumorigenic inflammation. However, at more advanced stages of carcinogenesis, tumor cells acquire inactivating mutations of TGFβ receptors and/or alteration of their signaling transduction pathway (reviewed in [12]) and become resistant to the negative regulatory effects of this cytokine. As a consequence, TGFβ switches from a tumor suppressor factor to a metastasis promoter and drives changes in the TME that finally sustain tumor growth [13]. Indeed, high levels of TGFβ in the TME enable cancer cells to escape the immune surveillance leading to an over-production of cytokines and chemokines that contribute to boosting chronic inflammation [12,13]. Moreover, TGFβ produced by cancer cells acts in a paracrine way on stromal cells stimulating the secretion of growth factors and mitogens, as the platelet-derived growth factor (PDGF), and driving the trans-differentiation of stromal progenitors, including resident fibroblasts, endothelial cells, preadipocytes, and bone marrow-derived mesenchymal stem cells (MSCs) into “activated fibroblast” [14,15,16]. These “activated fibroblasts”, collectively named CAFs, bear a myofibroblast-like phenotype, express higher levels of αSMA, collagen 11-α1 (COL11A1), PDGF receptor (PDGFR) α/β, and lower levels of caveolin-1 than normal fibroblasts, and acquire greater contractile and proliferating capacities [17].

**Figure 1:**
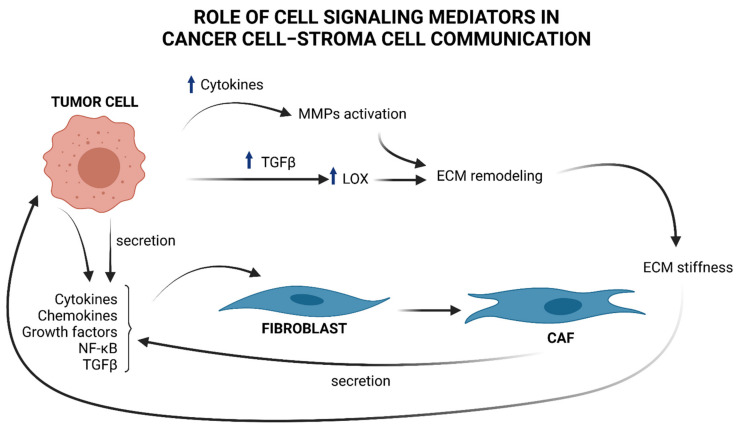
Brief overview of cell signaling mediators in cancer cell–stroma cell communication and ECM remodeling. Tumor cells produce cytokines, chemokines, NF-κB, and TGFβ, that “activate” stromal progenitors, such as fibroblasts, into CAFs and produce, in turn, cytokines and TGFβ, influencing tumor cell functions. TGFβ upregulation in cancer cells provokes an increment in LOX, which in turn affects ECM remodeling by changing the collagen organization and provoking ECM stiffness that induces the mechanical activation of latent TGFβ. An increased amount of cytokines by tumor cells induces MMPs activation that contributes to ECM remodeling and sustains tumor progress.

However, to date, specific markers to outline the molecular profile of CAFs are still lacking, making it difficult to better understand the biology of these heterogeneous classes of cells [18]. Once activated, CAFs themselves release TGFβ and feed an autocrine-positive signaling loop that sustains tumor progression by contributing to the generation of a permissive TME [19], as illustrated in Figure 1.

Under TGFβ stimulation, CAFs release high levels of cytokines and chemokines, contributing to attracting neutrophils, macrophages, lymphocytes, and natural killer (NK) cells at the site of the tumor lesion, thus boosting the inflammatory response that favors cancer progression [16]. One of the best characterized pro-tumorigenic cytokines is interleukin 6 (IL-6), known to be linked to increased risk of development of a large variety of cancers [20]. IL-6, secreted by CAFs, binds to its cognate receptor expressed by tumor cells and promotes their growth and the acquisition of an invasive phenotype by activating the IL-6–JAK–STAT axis and the notch pathway [21]. IL-6 secreted from cancer cells also acts in a paracrine manner to induce the differentiation of fibroblasts into CAFs. By these efferent (from tumor to stroma) and afferent (from stroma to tumor) signaling pathways, IL-6 is one of the molecules secreted in the TME that participates in the tumor–stroma cross-talk required for tumor growth [22].

Tumor necrosis factor-α (TNFα) is another cytokine secreted at high levels in the TME during the early stages of tumorigenesis that has an established role in chronic inflammation, angiogenesis, tissue remodeling, tumor growth, and metastasis [23]. Similarly to TGFβ, the role played by TNFα in cancer progression is both tumor-inhibiting and tumor-promoting, depending on the cell context and the cancer stage. Indeed, although high levels of TNFα inhibit tumor growth [24], secretion of low levels of TNFα by cancer cells in a B16 mouse melanoma model furthers the recruitment of infiltrating myeloid cells, promoting cancer vascularization and progression [25]. Furthermore, in a mouse xenograft model of ovarian carcinoma, TNFα receptor 1 (TNFR1) expression on CD4+ T cells is necessary for IL-17 secretion and myeloid cell recruitment in tumors, sustaining inflammation and cancer progression [26].

Apart from participating in the complex signaling between tumor and surrounding stroma, CAFs are also responsible for the deposition of ECM components (see Figure 1), a physiological process that becomes highly unregulated in the early stages of cancer [27]. This dysregulation compromises abundance, concentration, structure, and architecture of the ECM, affecting tumor establishment and progression. For example, deposition of collagen by CAFs, together with changes in the expression of the enzymes responsible for its remodeling, leads to an impairment of the organization of collagen fibers and results in increased stiffness of the ECM [28]. In breast cancer, the collagen fibers become linearized and perpendicularly oriented at the tumor boundary, thus favoring the migration and invasion of cancer cells [29]. The lysyl oxidases (LOX) enzymes are responsible for the cross-linking of collagen and elastin fibers. The expression of LOX is upregulated in many cancers by a mechanism that is, at least in part, TGFβ-driven [30]. An unbalanced enhancement in LOX expression and activity increases ECM stiffness that induces the mechanical activation of latent TGFβ and fuels the onset of a vicious circle that maintains a tumor-promoting inflammatory environment [28]. The activity of LOX enzymes is therefore not restricted to the shaping of the architecture and the mechanical functions of the ECM but is part of a complex network of bi-directional signaling within the matrix and different cells of the microenvironment that contributes to establish and maintain an inflamed, immune-suppressive, and pro-carcinogenic TME [30].

The activity of ECM-degrading proteases, for example, releases matrix-bound growth factors and cytokines, which can mediate the activation of downstream effectors, such as various oncogenic transcription factors [11,27].

Among these, NF-κB is a prototypical inflammatory mediator involved in tumorigenesis. NF-κB transient activation is tightly controlled under physiological conditions that promote inflammation, as an adaptive, physiological response. However, in cancer proinflammatory cytokines, oncogenic growth factors and tyrosine kinases promote the constitutive activation of NF-κB through autocrine and paracrine ways [31]. This persistent activation yields to the transcriptional regulation of various ECM components and ECM degrading enzymes, such as MMPs, whose expression is under the control of NF-κB [32].

To degrade the ECM, different types of cells which constitute the tumor mass, i.e., the actual cancer cells, activated fibroblasts, and macrophages, produce a large number of enzymes, belonging to the metzincin protease superfamily of zinc-endopeptidases, which include MMPs, also known as matrixins, a disintegrin and metalloproteinases (ADAMs), and ADAMs with thrombospondin motifs (ADAMTSs).

MMPs induced by pro- and inflammatory factors and regulated by NF-κB [32], as mentioned above, have an important role in sustaining all six major hallmarks of cancer [33] highlighted within the complexity of cancer biology: (1) sustaining proliferative signaling; (2) evasion of apoptosis; (3) angiogenesis and lymphangiogenesis; (4) invasion and metastasis; (5) reprogramming of energy metabolism; and (6) evasion of the immune response. Indeed, there is a close relationship between the release of cytokines and the specific ECM-degrading enzymes MMPs, as MMPs may play a non-proteolytic and non-ECM role in cell–cell communication [34] (Figure 1). For a deeper description of the role of specific MMPs regulated by cytokines, see Review [34].

## 3. Hyaluronan in TME: A Simple Extracellular Macromolecule with a High Impact in Tumor Progression

Solid tumors are characterized by a stiff, non-elastic matrix with an altered constitution of ECM components that results in increased interstitial fluid pressure and chemoresistance [35]. These concepts drive several studies towards strategies that target ECM compartments to develop therapeutic agents that facilitate drug perfusion and delivery by tumor cells. For instance, the use of hyaluronidase (HYAL) that degrades HA in breast cancer therapy has been approved by the FDA, demonstrating that HA has a critical role in cancer [36]. In addition, HA has also been considered a prognostic biomarker associated with a poor prognosis in patients with various types of cancers, such as breast, pancreatic, and ovarian, rendering HA an ECM macromolecule with a key role in cancer [6,37,38].

Alterations in HA metabolism, content, and deposition in inflammation [39] and cancer are usually related to induction or suppression of the HASs (the enzymes that synthesize HA) [33] and HYALS (the enzymes that degrade high molecular weight HA (HMWHA) into smaller fragments).

**Figure 2:**
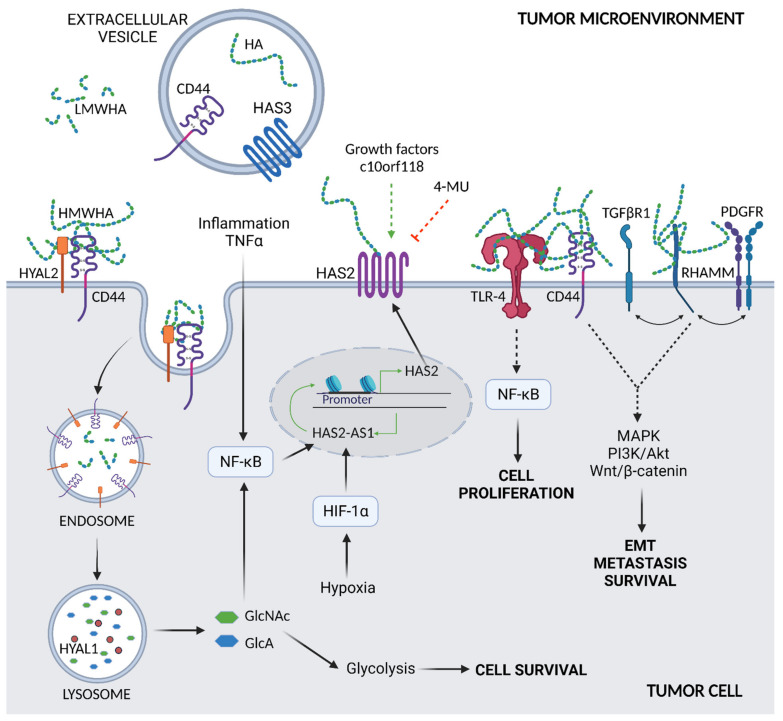
Overview of HA metabolism and interaction with membrane receptors. Synthesis of HA by HAS2, the main synthetic enzyme in cancer cells, is increased by inflammatory agents, such as TNFα, via the NF-κB signaling pathway. Induction of HAS2 also occurs due to various growth factors and the secreted protein c10orf118 in a not yet fully described mechanism. Pharmacological agents, such as 4-MU, inhibit HA synthesis and downregulate HAS2. A binding to HYAL2 and CD44 and endocytosis lead to the degradation of HMWHA; HYAL2 cuts HMWHA into fragments (LMWHA) at the plasma membrane which, in turn, are internalized into the cell via endocytosis and further degraded by HYAL1 in lysosomes. The produced sugars, GlcNAc and GlcA, may be recycled in cell energy metabolism, i.e., glycolysis, or control HAS2-AS1 expression via NF-κB. HAS2-AS1 is also regulated by hypoxia via HIF-1α. Tumor cells are secreted in tumor microenvironment extracellular vesicles that contain HA, HAS3, and CD44 that can be captured by target cells. The HA of the tumor microenvironment can bind to different cell membrane receptors. The HA–TLR-4 complex signals the NF-κB pathway, supporting cell proliferation, whereas HA-CD44 and HA-RHAMM interact with either TGFβRI and PDGFR, promoting EMT and cell survival and metastasis.

Differently from other GAGs that are synthesized in the Golgi, in mammals both synthesis and polymerization of HA occur on the plasma membrane by the three glycosyl-transferases HAS-1, -2, and 3, using UDP-GlcUA and UDP-GlcNAc as substrates [40,41]. These two sugar precursors allow HASs to work without ATP but exploiting the high energy content of the two UDP-sugar precursors [42]. Tumor cells can rapidly convert glucose into UDP-sugars, making available a great mass of HA substrates that enhance its synthesis [33]. Although all three HASs are found in various tumor cell types [43], HAS2 is the most efficient HA synthesizing enzyme [44] and its levels and activity are tightly regulated by post-translational modifications, such as ubiquitination, O-GlcNAcylation, and phosphorylation [45,46,47,48]. Secreted molecules in TME and inflammation can also modulate HAS2 activity. For instance, pro-inflammatory agents, such as IL-1, IL-6, and TNFα and β [49], as well as oxidized LDL [50] induce HAS2 activity and HA synthesis. Inhibition of HAS2 activity may be performed by pharmacological agents, such as 4-methylumbelliferone (4-MU) [51,52]. In particular, 4-MU is a chemopreventive and therapeutic agent that is used to treat prostate cancer [53]. Recently, it was demonstrated that treatment of estrogen receptor-positive breast cancer cells with 4-MU decreased cell migration, adhesion, and invasion [54]. These phenomena were accompanied by a reduced HAS2 expression and HA accumulation, induced HYALs, and substantial loss of the HA receptor CD44. An illustrated representation of HA synthesis and HAS2 induction is shown in Figure 2, indicating also that inflammation may induce HAS2 through the NF-κB pathway.

Recently, the natural antisense lncRNA hyaluronan synthase 2 antisense 1 (HAS2-AS1) was found to regulate HAS2 gene expression and thus HA synthesis [55,56]. HAS2-AS1 is essential not only in maintaining normal homeostasis, but it is also a pivotal factor in controlling the pathological conditions as it is an important stimulator for tumor cell proliferation and migration via, among all, HIF-1α and acting as a competing endogenous RNA for several micro RNAs [57,58,59,60].

The HA increment in the TME and the precancerous lesions is a result of the crosstalk between cancer and resident cells, thus many studies are focused on the mechanisms by which these cells interact. Recently, a new secreted protein produced in high amounts by breast cancer cells was found to be an important regulator of HAS2 in stromal cells. More specifically, in an in vitro model of co-culture of fibroblasts with breast cancer cells, the protein c10orf118 was shown to induce a significantly increased amount of secreted HA due to the increased levels of stromal HAS2 [61]. This protein belongs to the golgins family, vesicle tethering proteins that act selectively to tether transport vesicles at the Golgi apparatus. Indeed, the ortholog of c10orf118 in *C. elegans* co-localizes with Rab2 in the trans-Golgi network (TGN), regulating dense core vesicle maturation [62] and confirming c10orf118 involvement in the secretory pathway. The mechanism by which the HAS2 is overexpressed is still under investigation but seems to be mediated by the N-terminus part of this golgin.

One of the newly discovered crosstalks is the exchange of messages with vesicles that can transport various bioactive molecules, such as messenger RNA (mRNA), microRNAs, proteins, and bioactive lipids [63], as well as HA, CD44, and HAS3. The role of HAS3 in tumor progression is not related to its enzymatic activity, eventually altered by post-translational modifications, but more to its involvement in the induction of extracellular vesicles (EVs) shedding. Arasu et al. observed that increased amounts of EVs derived from GFP-HAS3 expressing metastatic melanoma cells induced HA secretion, proliferation, and invasion of target keratinocytes and primary melanoma cell line [64]. As described here, these HAS3-rich EVs contained high amounts of HA and, importantly, CD44 that participated in the regulation of EV binding to target cells [64]. In addition to cell functions’ associated genes induction, target cells may undergo EMT.

HA degradation is yielded by several HYALs: HYAL2 cuts HMWHA into fragments at the plasma membrane that, in turn, are internalized into the cell via endocytosis and further degraded by HYAL1 in lysosomes [65]. Recently, few new HA degrading enzymes have been described to have hyaluronidase activity, like the hyaluronan binding protein involved in hyaluronan depolymerization (HYBID) and the transmembrane protein 2 (TMEM2) [66], which is able to generate LMWHA. Intracellularly, HA is degraded by the action of HYALs, β-glucuronidase, and hexosaminidase, eventually generating free GlcUA and GlcNAc. At this point, GlcUA is converted into xylulose-5-phosphate that can be used by the cells either to sustain the pentose phosphate pathway or the non-oxidative part of the pentose phosphate pathway by increasing glycolysis and energy metabolism [67]. As cancer cells typically have high expression levels of HYALs [68], the active and sustained degradation of HA could contribute to metabolic reprogramming of cancer cells metabolism, de facto increasing the glycolytic rate to produce more ATP, and sustaining the pentose phosphate pathway to obtain reducing equivalents and ribose for anabolism [69]. Recently, it was demonstrated that the cancer resistance of naked-mole rats that were previously associated with an ultra-HMWHA [70] is accompanied by a low HYAL1 expression and overexpression of HYAL3 which, however, maintains an HMWHA in serum [71]. Despite the negative effect that the high quantity HYALs have in tumors, recent studies, including clinical trials, show that modified HYALs may be used in tumor treatments [35]. Indeed, as reported by Gao et al., on 29 June 2020, the FDA approved the subcutaneous injection of hyaluronidase-zzxf, together with pertuzumab and trastuzumab, for the treatment of patients who are HER2-positive in both early-stage and metastatic breast cancer [36]. Another study including a phase 2 clinical trial analyzed the collagens fragments and the proteoglycan (PG) versican in the human plasma of patients with a metastatic pancreatic ductal carcinoma treated with a human recombinant HYAL [72]. The treatment caused an improved overall survival, whereas it underlined the importance of ECM composition in the progression of both high- and low-levels HA-associated tumors [72].

The increased deposition of HA in tumors triggers diverse signaling events, mostly by interactions with cell surface receptors, among which the most relevant are CD44 and receptor for HA-mediated motility (RHAMM) [5,73]. Interestingly, HA size can influence receptor activation and downstream signaling [74].

CD44, a ubiquitous single-span transmembrane glycoprotein, is ubiquitously expressed in normal tissue. However, inflammation and cancer prevail over the expression of CD44 variants deriving from splicing events [75]. Interestingly, CD44 variants’ (i.e., CD44v and CD44s) expression in cancer have been reported as markers for poor prognosis [76] and regulators of EMT and plasticity of cancer cells [77]. There is also increasing evidence that CD44 can be considered a marker of cancer stem cells in breast, pancreas, and colorectal cancer, contributing to maintaining and initiating tumors [78,79,80]. HA–CD44 interaction involves an HA-binding motif on the CD44-N-terminal region, which is common to other HA-binding proteins, including RHAMM. HA–CD44 interaction triggers receptor clustering and interaction with several transmembrane proteins, including receptor tyrosine kinases, serine/threonine kinase receptors, TNFR-like receptors, G-protein coupled receptors, the Wnt receptor LRP5/6, CD147, and ATP-binding cassette transporters [77,81]. As a result, several signaling cascades are activated, among which are ERK1/2, Akt, Wnt/b-catenin, and focal adhesion kinase, thus stimulating oncogenic pathways and miRNA functions [82]. For a more extensive description of the role of HA-CD44 interaction, see Karousou E. et al. [7].

The second important HA receptor, RHAMM, is a coiled-coil type protein expressed both in the cytoplasm and on cell membranes, as well as in the cytoskeleton and nucleus. Like CD44, RHAMM undergoes alternative splicing as truncated isoforms were detected in tumor cells [83], and it has been reported to be highly expressed in several tumors, including breast, colon, brain, prostate, and endometrial [84,85,86,87]. After binding HA, RHAMM interacts with other receptors such as PDGFR, TGFβ receptor I (TGFβRI), and CD44 [75], thus inducing inflammatory pathways through ERK1/2 leading to cell migration, wound healing, tumorigenesis, and EMT. Intracellular RHAMM can also bind to the cytoskeleton contributing to microtubule-mediated cell polarity and motility. Finally, nuclear RHAMM binds mitogen-activated protein kinase (MAPK), which mediates the activation of MMP-9, inducing inflammation and cell migration [88]. For a more detailed analysis of the HA/RHAMM role, see Kouvidi K. et al. [89].

Another interesting HA-binding protein is TNF-stimulated gene 6 (TSG-6), which yields the formation of cross-links between HA and the heavy chains of the serine protease inhibitor inter-alpha-inhibitor (IαI), thus stabilizing the structural integrity of ECM. In general, TSG-6 is not a constitutively expressed protein in normal adult tissues, but its expression is upregulated during inflammation and cancer progression [90,91].

The Toll-like receptors (TLRs) family is a group of membrane receptors found mainly in the immune system and correlated to inflammation during viral infection and tumorigenesis [92,93,94]. Among other receptors, HA binds the TLR4 promoting tumor growth and differentiation. Recent studies showed that in colon tumorigenesis, HA interacts with both CD44 and the abundant TLR4, promoting the growth of tumor grafts in mice [95]. Another study on glioblastoma stem-like cells demonstrates that HA triggers the TLR4–NF-κB pathway provoking the differentiation of cells that, in this way, maintain the increased proliferation and tumorigenic capacity [96].

## 4. Extracellular Chaperones in the TME: Focus on Their Roles in Cancer-Related Inflammation and ECM Remodeling

The protein homeostasis of cancer cells is challenged by an increased rate of protein synthesis that is required to face the anabolic demand of fast-growing and proliferating cells. Oxygen and nutrients limitations drive changes in the metabolism of cancer cells that contribute to the maintenance of an acidified, oxidized, and inflamed TME favoring the unfolding of many extracellular proteins [97].

As a consequence, cancer cells upregulate the activity of their protein quality systems that collectively constitute the proteostasis network in the attempt to reduce the proteotoxic stress and keep the proteome as stable and functional as possible [98].

Molecular chaperones are first-line players of the proteostasis network due to their ubiquitous expression and their ability to stabilize proteins in their native conformation, re-fold denatured polypeptides, inhibit unfolded protein aggregation, and direct misfolded proteins to proteasomal and autophagic degradation [99,100]. The wide and heterogeneous family of molecular chaperones comprises, among others, the heat shock proteins (HSPs), a family of stress-proteins that are induced in response to physical, chemical, and biological insults to support cell survival [101]. According to their molecular weight, HSPs are currently classified into six groups. HSP100s, HSP90s, HSP70s, and HSP60s use the energy derived from ATP hydrolysis to assist protein folding. HSP40s and other small HSPs do not possess ATPase activity and act as a co-chaperone to HSP70s, binding to unfolded proteins preventing their aggregation and eventually delivering those irreparably damaged to the protein degradation systems [102].

It is now widely accepted that many HSPs, initially classified as intracellular proteins, can reach the extracellular space through various mechanisms, including exocytosis of lysosomal vesicles, translocation through the plasma membrane, and release of extracellular vesicles as exosomes [103,104,105,106].

The function of extracellular HSPs (eHSP) in the TME is not limited to the canonical function of stress proteins that counteract protein unfolding (reviewed in Richter K. et al.) [107]. More recent studies highlighted that eHSPs take part in the complex signaling network that influences the fate of cancer cells with different outcomes depending on the stage of the pathology [101]. They counteract tumor growth due to their ability to bind antigenic peptides and activate anti-tumor innate immunity [108], thus promoting immune surveillance and inhibiting inflammation [109]. However, they favor tumor progression and metastasis formation by sustaining cancer proliferation, cell migration and invasion, neo-angiogenesis, and immune escape [101].

The eHSPs interact directly with cell surface receptors and consequently engage intracellular signaling pathways that influence cell behavior [101]. Low-density lipoprotein receptor-related protein 1 (LRP1) is a common receptor for many extracellular chaperones, both in normal and cancer cells where its expression is often upregulated [110]. eHSP90 following LRP1 binding activates both AKT and NF-κB signaling, promoting cell proliferation, EMT, and ECM remodeling [111,112]. Similarly, various eHSPs activate Toll-like receptors (TLRs), induce pro-inflammatory signaling cascades through NF-κB and ERK activation, and promote tumor establishment releasing pro-inflammatory, anti-apoptotic, proliferative, and pro-fibrogenic signals on cancer cells and TME cells [113].

Many ECM-degrading enzymes depend on eHSPs binding for their activity and stability. For instance, in breast cancer, eHSP90α mediates the proteolytic activation of MMP2 in cooperation with eHSP70 and other co-chaperones [114]. eHSP90α interacts with lysyl oxidase-like 2 (LOXL2) and allows the latter to assume its functional conformation favoring the migration of breast cancer cells [115] and with tissue plasminogen activator (tPA), favoring the conversion of plasminogen in plasmin, a process that favors cancer invasion [116]. In breast cancer cells, both HSP90α and HSP90β are closely associated in a complex with fibronectin, participating in its assembly and turnover [117,118].

### Clusterin: A Prominent Extracellular Chaperone in the TME

Clusterin (CLU) is a heterodimeric glycoprotein of 80 kDa, secreted in almost all biological fluids, first identified in ram rete testis where it showed signs of clustering with rat sertoli cells and erythrocytes [119]. CLU is involved in a plethora of fundamental cellular processes and has been recently proposed as one of the most prominent extracellular ATP-independent chaperones, functionally related to the sHSPs, exhibiting anti-inflammatory functions [120,121].

Unlike other well-known HSPs, CLU is synthesized by ribosomes attached to the ER and reaches the outside of the cells by the canonical ER–Golgi route [122]. Once in the extracellular compartment, CLU binds to a variety of structurally unrelated client molecules through a dynamic disordered region containing amphipathic α-helices [123]. Of note, CLU not only prevents their aggregation but also commits them to lysosomal-degradation through endocytosis mediated by surface receptors [124]. A more recent study identified heparan sulfate (HS) as the main clearance receptor involved in CLU-client protein complex endocytosis and subsequent lysosomal degradation. Of note, the CLU-HS pathway may represent a ubiquitous and versatile degradation pathway for different extracellular damaged or unwanted ligands [125]. By these combinations of binding and scavenging properties, CLU participates in the reestablishment of the extracellular protein homeostasis [126].

According to its function, CLU has been involved in many diseases that are accompanied by an impairment in protein synthesis and protein quality maintenance, including neurodegeneration, inflammatory diseases, and cancer [127,128,129,130]. Many cancer- and inflammation-related transcription factors, as NF-κB, HIF1α, TGFβ, TNFα are involved in the transcriptional regulation of CLU during cancer progression [131]. CLU is down-regulated in the vast majority of primary and naïve cancers in comparison to normal tissue [130,131,132] by epigenetic mechanisms that include CpG islands hypermethylation and histone tail modification [133,134,135,136,137,138]. Conversely, CLU expression is upregulated in the late stages of carcinogenesis, especially in niches of cancer cells resistant to chemotherapy or hormonal therapy [139].

In line with the observations reported above, the abrogation of CLU expression in mouse models of neuroblastoma, prostate, lung, and skin carcinogenesis leads to a quicker progression of the neoplasia toward the invasive phenotype [140,141,142,143], while targeting CLU with siRNA or antisense oligonucleotides enhances the cytotoxicity of chemotherapeutic drugs, ionizing radiation, and hormonal therapy [144,145].

**Figure 3:**
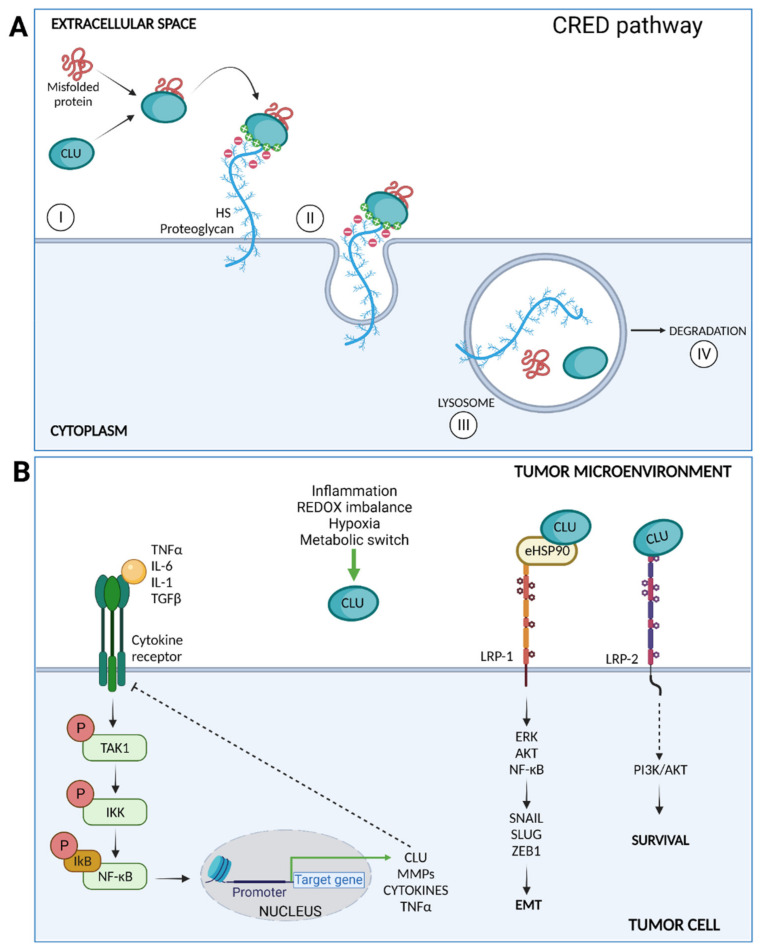
(**A**) CLU mediates clearance of misfolded proteins through the chaperone- and receptor-mediated extracellular protein degradation (CRED) pathway. CLU binds a misfolded protein and then interacts with the HS chains membrane PGs by electrostatic interactions (I); the CLU–client protein-receptor is endocytosed (II) and delivered to the lysosome (III) for intracellular degradation (IV). (**B**) Intracellular signaling pathways triggered by CLU after surface receptors binding. CLU inhibits the TAK1– NF-κB signaling axis triggered by inflammatory cytokines. CLU facilitates eHSP90 binding to LRP1, enhancing the activation of ERK, AKT, and NF-κB and promoting EMT. Finally, CLU binds LRP2 and promotes cell survival through PI3K/AKT activation.

The apparent contradictory tumor limiting and promoting functions of CLU represents instead the two sides of the same coin, which are consistent with a cytoprotective role exerted by the protein, both under normal and pathological conditions. Actually, at early stages of carcinogenesis, CLU expression is required to counteract the proteotoxic stress after increased anabolism that in turn drives genomic instability onset. Differently, under the selective pressure of cytotoxic therapies and altered signal transduction, CLU is over-expressed by resistant cells and favors the onset of a permissive tumor microenvironment. Some of the functions played by CLU in the inflamed TME are illustrated in Figure 3.

In this scenario, different studies have shown that increased levels of CLU correlate with the induction of EMT in various cancer cell lines [146,147,148,149,150]. Coherently, CLU silencing is accompanied by an increase in epithelial markers as E-cadherin and junctional zona occludens-1 (ZO-1) concomitantly with the loss of the mesenchymal markers fibronectin, N-cadherin, and vimentin [146,147,148,149,150]. CLU is one of the most significantly upregulated genes in the murine breast cancer cell line BRI-JM01, undergoing TGFβ-induced EMT, where its neutralization with an anti-CLU antibody blocks the acquisition of the mesenchymal phenotype induced by TGFβ [146]. The positive modulatory effects of TGFβ on CLU transcription is mediated by Twist-1, an EMT-promoting transcription factor [148]. In hepatocellular carcinoma cells, CLU silencing leads to a reduction in Smad3 phosphorylation in concomitance with the inhibition of EMT markers [149]. This finding suggests that CLU in the meantime is a transcriptional target of TGFβ and positively modulates the TGFβ signaling transduction through the canonical Smad pathway. In breast cancer cells, CLU favors the binding of eHSP-90α to the surface receptor LRP-1 and potentiates the transduction of EMT-promoting signaling [150].

In prostate, breast, and colon cancer, the expression of CLU changes with disease progression both in the epithelial cancer cells and in the surrounding stromal tissue [151,152,153]. Of note, only clusterin stromal staining significantly correlates with disease recurrence and clinical outcome following surgical intervention or chemotherapy [153,154].

These results highlight the concept that stromal non-cancer cells contribute to shaping the biological behavior of cancer cells, strongly suggesting a role of CLU in ECM remodeling. Disappointingly, so far only a limited number of studies have specifically addressed this issue. In vitro studies conducted in breast, renal, nasopharyngeal, and hepatocellular cancer cell lines found that MMP-9 and MMP-2 expression is reduced following CLU silencing, concomitantly with a decrease in cell motility and invasion [155,156,157,158].

Collectively, all these studies limited their investigation to evaluating the changes of MMPs expression in response to CLU gene manipulation (up- or down-regulation), without paralleling gene expression data with measures of the enzymatic activity. In contrast, experiments performed in normal kidney cells, in neutrophils, and in breast cancer cell line MCF-7 found that CLU binds to and inhibits the activity of MT6-MMP, a member of the membrane-type MMPs [159]. An inhibiting effect of CLU on MMPs activity was also confirmed in the context of dry eye syndrome, an inflammatory disease caused by inadequate hydration and lubrication of the ocular surface. In a mouse model of dry eye disease, the induction of the pathology is accompanied by a decrease in CLU expression and a concomitant increase in MMPs expression. The authors demonstrated that CLU prevented stress-induced MMP-9 aggregation and activation, both inside and outside human epithelial cells, by direct binding with the catalytic domain. The inhibition of the enzymatic activity was also demonstrated for MMP-2, MMP-3, and MMP-7 [160]. In addition, TNFα stimulation of corneal cultured cells induced CLU upregulation, along with a decrease in MMP-9 expression, while the topical administration of CLU protected the ocular surface barrier from functional disruption after dry eye induction in a mouse model [161]. These data suggest a role for CLU in preventing the inflammatory status of the eye maintaining the normal fluid/epithelial interface homeostasis.

In support of the anti-inflammatory function of CLU, our laboratory recently showed that CLU limits NF-κB hyperactivation in prostate cancer cells and pre-clinical models of prostate cancer [162]. More precisely, CLU overexpression in PC3 cells is accompanied by a significant reduction of the phosphorylation of the p65 subunit of NF-κB. This led to reduced nuclear translocation of NF-κB along with a reduction of MMP-2 and MMP-9, while CLU silencing in the same cells produced opposite effects. In line with these data, we found a significant increase of MMP-2 and MMP-9 gelatinase activity in a transgenic murine model of prostate cancer knock-out for CLU (TRAMP/CLUKO) compared with age-matched prostate cancer-prone mice (TRAMP) expressing CLU [162]. Recently, another study showed that CLU inactivation leads to the constitutive activation of the TAK1–NF-κB signaling axis in experimental models of non-small cell lung cancer and promotes EMT in vitro and metastasis formation in vivo [141].

Altogether, these data suggest that CLU expression may be required to limit the excessive activation of NF-κB that occurs during chronic inflammation associated with tumor growth. The inhibition of the MMPs activity, therefore, would be part of an array of anti-inflammatory properties that CLU shares with other members of the sHSPs. In this context, the abrogation of CLU expression at early stages of carcinogenesis would fail to dampen NF-κB activity resulting in the generation of a TME conducive to the process of tumor invasion.

## 5. Conclusions

Normal cells evolve to a neoplastic state through a complex multi-step process that allows them to acquire the ability to sustain uncontrolled proliferation. These general capabilities were initially summarized in six “hallmarks”, according to Hanahan [163]. However, the last two decades of active research in the field of tumor biology highlighted that cancer cells are not simply “insulae” of proliferating cells and that the TME comprises heterogeneous cell types, including immune cells and CAF, as well as structural elements, such as collagen and hyaluronan that constitute the ECM [2].

We showed in this review that ECM and cellular heterogeneity are reciprocally linked, and it is the interplay between stroma and cancer that drives the progression of the disease toward an increasingly aggressive and invasive phenotype. In this view, chronic inflammation and loss of ECM homeostasis represent enabling characteristics, if not hallmarks, that favor tumor growth and progression. Even more recently, altered protein homeostasis has been proposed to be a new distinguishing trait of tumors, which participate in TME shaping and ECM remodeling. Indeed, cancers upregulate a plethora of molecular chaperones to adapt to unfavorable environmental conditions, such as inflammation, nutrient deprivation, hypoxia, and altered ECM deposition.

Although inflammatory molecules, ECM components, and extracellular chaperones derive from three well-defined and independent processes, altogether they influence each other and modulate the behavior of the heterogeneous cellular components of TME by triggering pleiotropic and redundant signaling pathways by interacting with transmembrane proteins.

In conclusion, quantitative compositional and physical changes occurring in the TME synergistically contribute to tumor heterogeneity and influence various aspects of stroma–tumor coevolution. However, many mechanisms remain poorly understood in part due to a lack of experimental model systems that can recapitulate both cellular and ECM complexity. Therefore, the development of suitable, affordable, and sufficiently robust engineered models mimicking the TME complexity are urgently needed to determine the cause–effect relationship between inflammation, ECM changes, and altered proteostasis described in this review.

## Data Availability

Figures presented in this review were created with BioRender.com.

## Abbreviations

ADAMS	a disintegrin and metalloproteinases
ADAMTS	ADAMs with thrombospondin motifs
CAFs	cancer-associated fibroblasts
CLU	clusterin
ECM	extracellular matrix
EMT	endothelial-to-mesenchymal transition
EVs	extracellular vesicles
GAG	glycosaminoglycans
GlcA	(β, 1-4)-glucuronic acid
GlcNAc	(β, 1-3)-N-acetyl-glucosamine
HA	hyaluronan
HAS	hyaluronan synthase
HAS2-AS1	hyaluronan synthase 2 antisense 1
HMWHA	high molecular weight hyaluronan
HS	heparan sulfate
HSPs	heat shock proteins
HYAL	hyaluronidase
IL-6	inteleukin-6
LMWHA	low molecular weight hyaluronan
LOX	lysyl oxidases
LRP1	low density lipoprotein receptor-related protein 1
MMPS	matrix metalloproteases
MT1-MMP	membrane type 1 MMP
PGs	proteoglycans
PDGF	platelet-derived growth factor
PDGFR1	PDGF receptor 1
RHAMM	receptor for HA-mediated motility
TGFβ	transforming growth factor beta
TLR	Toll-like receptor
TME	tumor microenvironment
TNFα	tumor necrosis factor alpha
VEGF	vascular endothelial growth factor
VEGFR	VEGF receptor
